# A study of adverse maternal-foetal outcomes in nephrotic syndrome combined with preeclampsia

**DOI:** 10.1186/s12884-023-06073-8

**Published:** 2023-11-07

**Authors:** Dong Li, Minyi Zhang, Shuxiu Xu, Ziwei Bian, Xiaoli Huang, Guifang Hu, Jing Li

**Affiliations:** 1 Department of Epidemiology, School of Public Health, Southern Medical University, Guangzhou, 510515 Guangdong China; 2 Department of Obstetrics and Gynecology, Nanfang Hospital, Guangzhou, Guangdong China

**Keywords:** Nephrotic syndrome with preeclampsia, Nephrotic syndrome, Pregnancy outcomes, Prevention

## Abstract

**Background:**

Although the majority of pregnancies with preeclampsia are characterised by elevated blood pressure, preeclampsia is often associated with nephrotic syndrome with similar symptoms such as high proteinuria and bilateral lower limb oedema. In this study, we compared the maternal–foetal outcomes of pregnant women with preeclampsia in a population with nephrotic syndrome and explored the factors that contribute to the corresponding outcomes and disease development.

**Methods:**

A total of 90 pregnant women were included in this study, of whom 30 had nephrotic syndrome and were diagnosed with preeclampsia during pregnancy, and 60 had nephrotic syndrome alone. Descriptive statistical analyses of baseline data were performed to analyse the effect of combined preeclampsia on maternal and foetal pregnancy outcomes using unadjusted and adjusted logistic regression models.

**Results:**

In this study, the baseline data of the two study populations demonstrated no differences except for the history of caesarean section and 24-h proteinuria results, which were significantly different (*P* < 0.05). The risk of preterm birth in the nephrotic syndrome with preeclampsia group was 8.25 (95% CI:3.041–22.084 *P* < 0.05); for a low birth weight, the risk was 6.00 (95% CI:2.302–15.638 *P* < 0.05); for foetal distress,the risk was 5.667 (95% CI:2.070–15.514* P* < 0.05); and the risk of foetal birth restriction was 7.429 (95% CI: 2.642–20.885 *P* < 0.05). A risk-based analysis of adverse maternal outcomes yielded a risk of miscarriage of 2.200 (95% CI: 0.584–8.291; *P* > 0.05). After adjusting the model for each outcome, significant risks of preterm labour, foetal birth restriction, and low birth weight were revealed (*P* < 0.05).

**Conclusion:**

Combined preeclampsia has a significantly higher risk of adverse pregnancy outcomes for the foetus.Therefore, the prevention and control of eclampsia in pregnant women should be improved to ensure maternal and neonatal health.

## Introduction

Nephrotic Syndrome (NS) is a disease caused by a lack of kidney function [1]. Although the causes of NS are unclear, some scholars believe that the main factor involves the disruption of the immune system [2]. This damages the podocytes, which affects the osmotic function and ultimately the evolution of NS, which in turn causes kidney dysfunction and affects the normal metabolism [3, 4]. As a result, the patient can experience a series of symptoms, such as oedema, proteinuria, hypoproteinaemia, and hypercholesterolaemia [3]. Some womenwith NS demonstrate no evidence of kidney dysfunction. Studies have shown that women with normal NS pregnancies who do not have significant hypertension or renal insufficiency have better maternal and foetal outcomes [5]. Preeclampsia(PE), a common condition in pregnancy belonging to the "grand mal" group of obstetric syndromes, affects approximately 4.6% of all pregnancies worldwide [6, 7]. Research on its pathogenesis has identified a link with uteroplacental ischaemia [6, 8]. Preeclampsia usually develops at approximately 20 weeks of gestation and is characterised by persistent hypertension, high proteinuria,and oedema [9, 10]. If maternal preeclampsia is not diagnosed early and managed effectively, the risks to the mother and foetus are enormous and irreversible [11]. Therefore, early prevention and management of maternal preeclampsia are essential for both the mother and foetus. Because of the similarity of symptoms to NS, the diagnosis and prevention of both are often closely linked [5, 12, 13]. Studies on the pregnancy outcomes of NS induced by PE during pregnancy are clear; however, few studies have examined the outcomes of PE during pregnancy in people with NS. Therefore, here we aimed to analyse the harmful effects of PE on maternal and foetal outcomes in NS by examining the effects of combined PE during pregnancy on maternal and foetal outcomes in the NS population.

## Method

### Study population

**Fig. 1:**
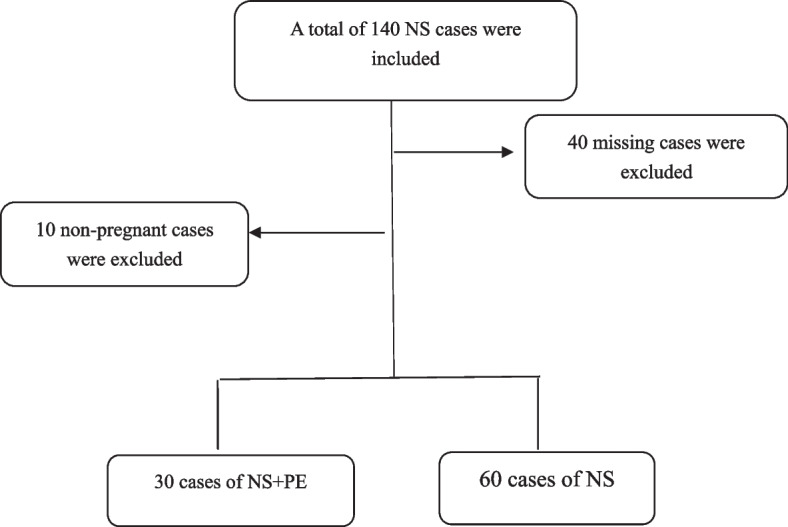
Case selection flowchart

Nanfang Hospital is a comprehensive tertiary hospital in South China, and pregnant patients with NS who attended our hospital in the past ten years, from 2012 to 2021, were included in this study. NS was diagnosed by the Clinical Guidelines for the Diagnosis of Kidney Diseases (2012): patients with urine protein levels persistently higher than 300 mg/d with symptoms such as hyperproteinemia and edema or those diagnosed by renal biopsy. Preeclampsia was mainly diagnosed by medical professionals according to the Guidelines for the Diagnosis and Treatment of Hypertensive Diseases in Pregnancy (2015). The specific data screening process is shown in Fig. 1. From 2012 to 2021,140 cases of NS were screened in the case management centre of the Southern Hospital. After secondary screening, 90 cases with complete data were retained, including 30 cases of nephrotic syndrome combined with preeclampsia and 60 cases of NS. Therefore, this retrospective study aimed to investigate the effects of PE during pregnancy on the mother and the foetus in patients with NS, as well as the factors affecting the outcomes of the pregnancy.

### Identification of NS and preeclampsia populations and outcome types

Of all patients with NS, some underwent kidney biopsy and were diagnosed with NS, while others were diagnosed based on renal function tests in our clinic and by persistent 24-h urine protein levels > 300 mg/d. Preeclampsia occurs mainly during pregnancy and is diagnosed by persistent blood pressure ≥ 140/90 mmHg, 24-h urine protein levels ≥ 300 mg/dl, or urine protein-to-creatinine ratios ≥ 0.3 mg/mmol. The patients were divided into two main groups: the NS with PE group and the NS group. The main maternal and foetal outcomes were as follows: preterm birth ( i e,delivery before 37 weeks of gestation), low birth weight ( i e,birth weight < 2500 g), fetal distress ( i e,insufficient oxygenation of the foetus during gestation or delivery), fetal birth restriction ( i e,ultrasound weight or abdominal circumference of the foetus lower than the 10th percentile of the appropriate gestational age), stillbirth, and miscarriage; and maternal outcomes were: postpartum haemorrhage, and haemorrhagic tubal inflammation.

### Statistical analyses

The data were statistically analysed using SPSS version 26.0. The data distributions were examined using P-P plots to test whether they were normally distributed. Data that conformed to a normal distribution are expressed as mean ± standard deviation; data that did not conform to normal distribution are expressed as quartiles. Normally distributed continuous variables were compared using analysis of variance (ANOVA); enumerated variables are expressed as ratios, and group differences were compared using Pearson's chi-square test or Fisher's exact test; data that did not conform to a normal distribution were analysed using the Mann–Whitney U test. A logistic regression test was used to further analyse the odds ratios (ORs), 95% confidence intervals (CIs) and p-values between the two groups to determine the risk of combined PE. Finally, the multivariate logistic regression model was used to further clarify the riskiness of the combined PE group to the NS group by adjusting for possible confounders. Among the risk model adjustment factors, the main ones were: 24H urine protein, pregnancy frequency, history of cesarean section, total calcium, uric acid, and serum triglycerides.

## Results

1. General information comparing results: (Indicators expressed as means were compared using one-way ANOVA; those expressed as quartiles were analysed using the Mann–Whitney U test; and rate information was analysed using chi-square analysis).

As shown in Table 1, the general data analysed included age, height, weight, body mass index(BMI), history of caesarean section, history of induction of labour, number of pregnancies, number of deliveries. BMI was obtained by dividing the weight at admission (Kg) by the square of the height (m). As shown in Table 1: the incidence of cesarean section in the combined group was significantly higher than that in the regular NS group. In addition, the number of pregnancies and 24-H urine protein levels were also significantly higher in the combined group than in the NS group (*P* < 0.05).

**Table 1:** Comparative results of baseline data

Characteristics	NS + PE(30)	NS (60)	*P*
Height(m)	1.59 $\pm 0.05$	1.59 $\pm 0.05$	0.77
Age(y)	34.5 $\pm 5.53$	33.3 $\pm 4.66$	0.375
BMI(Kg/m2)	27.25(25.50, 30.50)	26.77(25.00, 29.08)	0.22
Preconception weight(Kg)	55.50(49.63, 61.25)	54.00(50.00, 62.00)	0.32
Admission weight(Kg)	70.50(63.63, 75.75)	67.10(62.63, 73.00)	0.11
History of caesarean section	22(73.33%)	25(41.67%)	0.005
History of induction of labour	3(10%)	5(8.33%)	0.79
Number of pregnancies	2.83 $\pm 1.42$	2.18 $\pm 1.17$	0.032
Number of fetuses	1.70 $\pm 0.79$	1.43 $\pm 0.56$	0.14
First birth 24-H urine protein(mg)	12(40%) 5.43(1.98, 10.60)	30(50%) 0.88(0.25,4.76)	0.37 0.001

2 Comparison of clinical indicators during pregnancy between the combined group and the nephrotic syndrome group

(Indicators expressed as means were compared using one-way ANOVA; those expressed as quartiles were analysed using the Mann–Whitney U test; and rate information was analysed using chi-square analysis).

As shown in Table 2, the comparative analysis of the clinical parameters during pregnancy revealed that the triglyceride levels in the combined group were more abnormal than those in the NS group, and the difference was statistically significant (*p* < 0.05). In the comparative analysis of the six items of body ions, total calcium levels were different, and the total calcium level of the combined group was higher than that of the NS group (*P* < 0.05). In the analysis of the five renal function items, the uric acid level of the combined group was significantly higher than that of the NS group(*P* < 0.05). In the comparison of the routine blood tests, only the red blood cells (RBC) and the percentage of neutrophils (MONO%) showed a difference (*P* < 0.05).

**Table 2:** Comparative results of indicators between the combined group and the NS group

Variables	NS + PE(30)	NS (60)	*P*
serum triglyceride	3.27(2.44,5.46)	1.81(1.18,3.54)	0.001
low-density lipoprotein (LDL)	4.27(2.91,5.40)	3.47(2.90,4.28)	0.064
high-density lipoprotein (HDL)	1.78(1.37,2.06)	1.63(1.43,2.06)	0.804
total cholesterol	6.74(5.44,8.71)	6.12(4.82,7.04)	0.023
24H urine protein	5.43(1.98,10.60)	0.88(0.25,10.76)	0.001
International Standard Ratio of Coagulation	0.88(0.82,0.93)	0.89(0.85,0.96)	0.085
Activated partial thromboplastin time(APTT)	27.63 $\pm 4.39$	26.90 $\pm 3.87$	0.43
plasma prothrombin time(PT)	10.10(9.50, 10.58)	10.30(9.80,10.70)	0.15
ion hexa			
K +	4.35 $\pm 0.39$	4.05 $\pm 0.37$	0.001
NA +	137.5(135,139)	138(137,139.75)	0.063
**CL-**	105.58 $\pm 3.12$	105.61 $\pm 2.41$	0.96
Total Ca	2.03(1.93,2.24)	2.22(2.15,2.23_	0.000
Inorganic-P	1.32(1.18,1.51)	1.23(1.07,1.41)	0.112
Mg	0.87(0.76,1.56)	0.78(0.74,0.84)	0.006
renal function			
Total Co2	18.47 $\pm 4.40$	19.86 $\pm 2.58$	0.062
urea	5.85(4.40,8.03)	4.15(3.15,5.78)	0.001
uric acid	451.50(381.50,595.50)	376.00(316.75,458.50)	0.002
creatinine	74.00(57.75,101.25)	58.50(45.00,90.25)	0.016
liver function			
ALT	13.00(10.00,20.50)	9.00(7.00,12.00)	0.001
AST	19.50(15.75,25.75)	15.00(12.00,18.00)	0.000
Total protein (TP)	54.35(49.75,60.83)	60.95(55.65,63.93)	0.005
ALB	28.95(23.00,32.35)	34.70(32.50,37.60)	0.000
Globulin (G)	26.17 $\pm 4.26$	25.67 $\pm 4.15$	0.593
ALB/G	1.10(0.98,1.30)	1.40(1.20,1.50)	0.000
total bilirubin	3.20(2.38,5.33)	3.75(2.93,5.28)	0.433
direct bilirubin	1.60(0.90,2.50)	1.65(1.30,2.30)	0.643
indirect bilirubin	1.90(1.08,2.88)	2.05(1.35,3.00)	0.581
routine blood test			
RBC	3.74(3.48,4.38)	3.95(3.50, 4.29)	0.59
monocyte count(MONO)	0.44(0.35, 0.68)	0.58(0.45, 0.76)	0.031
MONO%	5.25%(4.00%, 6.38%)	6.15%(5.00%, 7.60%)	0.025

3 Comparison of pregnancy outcomes between the combined and NS group (Differences between groups were compared using chi-square analysis)

As shown in Table 3, 90 pregnancies demonstrates adverse maternal outcomes, including 25 cases of postpartum haemorrhage of which 10 were in the combined group and 15 were in the NS group (33.3%:25%), and 3 cases of haemorrhagic salpingitis, with 2 in the combined group and 1 in the NS group (6.7%:1.7%). The two groups had no statistically significant differences (*P* > 0.05).

**Table 3:** Comparative results of pregnancy outcomes

Pregnancy outcomes	NS + PE(30)	NS (60)	*P*
Maternal outcomes			
postpartum haemorrhage	10(33.3%)	15(25%)	0.41
Haemorrhagic salpingitis	2(6.7%)	1(1.7%)	0.21
Fetal outcomes			
premature birth	22(73.3%)	15(25%)	< 0.001
abortion	5 (16.7%)	5 (8.3%)	0.24
stillbirth	4 (6.7%)	5 (8.3%)	0.46
Low body weight(< 2500 g)	20 (66.6%)	15 (25%)	< 0.001
fetal distress	15 (50%)	9 (15%)	< 0.001
Fetal birth restriction	16 (53.3%)	8 (13.3%)	< 0.001

In a comparison of foetal outcomes between the combined and NS groups, found 37 cases of premature birth (22 [73.3%] in the combined group and 15 [25%] in the NS group); 10 cases of miscarriage (5[16.7%] in the combined group and 5 [8.3%] in the NS group); 9 cases of stillbirth (4 [6.7%] in the combined group and 5 [8.3%] in the NS group; 35 cases of low birth weight ( 20 cases in the combined group [66.6%] and 15 cases in the NS group [25%]); 24 cases of foetal distress ( 15 [50%] in the combined group and 9 [15%] in the NS group; and 24 cases of foetal birth restriction (16 [53.3%]in the combined group and 8 [13.3%] in the NS group). Among these outcomes, there were significant differences in preterm birth, miscarriage, low birth weight, fetal distress, and fetal birth restriction between the two groups. (*P* < 0.05), whereas stillbirth was not statistically significant (*P* > 0.05).

As shown in Table 4, the maternal risk before and after combined PE was not significant. For the foetus, PE presented a higher risk in the univariate analyses of all adverse outcomes. However, except for preterm labour, these were not statistically significant in subsequent multifactorial analyses. In the unadjusted one-way analysis for preterm labour, PE showed a higher risk (OR = 8.25 [3.04–22.38]; *P* < 0.05). After subsequent adjustment for the single factor, combined PE still presents a high risk of preterm foetal outcomes: OR = 6.74 (2.41–18.81) (*P* < 0.001). This demonstrates that combined PE significantly increased the risk of preterm birth.

**Table 4:** Logistic regression analyses of factors affecting primary outcomes. (Analysis of the risk of PE on outcome using logistic regression)

variables	Univariable analysis OR(95%CI)	*P*	Multivariable analysis OR(95%CI)	*P*
premature				
PE	8.25 (3.04–22.38)	< 0.001	6.74 (2.41–18.81)	< 0.001
fetal distress				
PE	5.67 (2.07–15.51)	0.001	1.75 (0.50–6.10)	0.38
Low birth weight babies				
PE	6.00 (2.30–15.64)	< 0.001	2.07 (0.66–6.46)	0.21
Fetal birth restriction				
PE	7.43 (2.64–20.88)	< 0.001	2.85(0.79–10.32)	0.11
abortion				
PE	2.20 (0.58–8.29)	0.22	2.38 (0.32–17.47)	0.39
postpartum haemorrhage				
PE	1.50 (0.58–3.91)	0.41	1.05 (0.37–3.04)	0.92
Haemorrhagic tubulitis				
**PE**	**4.21 (0.37–48.46)**	**0.25**	**2.41 (0.12–47.67)**	**0.57**

4 Association of combined PE with adverse pregnancy outcomes: (Logistic regression was used to analyse the risk before and after adjustment)

The common clinical adverse pregnancy outcomes for the foetus consist of: preterm delivery, foetal birth restriction, foetal distress, low birth weight, and miscarriage. Meanwhile, the more prevalent adverse maternal outcomes, postpartum haemorrhage and haemorrhagic tubulitis, were selected to analyse the risk of PE in the occurrence of these endpoints. The results of the model construction are shown in Table 5, the maternal outcomes haemorrhagic salpingitis and postpartum haemorrhage had no statistically significant differences before and after adjustment of the model (*P* > 0.05). For foetal outcomes, preterm birth, fetal birth restriction, and low birth weight remained different after multiple adjustments to the model, with ORs of 7.26 (2.54–20.70), 4.07 (1.32–12.51), and 3.42 (1.21–9.69), respectively. For foetal distress, models 1 and 2 demonstrated a statistically significant differences (*P* < 0.05), which ceased to be statistically significant after the addition of total calcium, uric acid, and triglycerides levels and RBC count in model 3. Regarding the adverse outcome of miscarriage, the unadjusted model showed no difference(*P* > 0.05). However, after adjusting for some of the factors in model 2, differences appeared; and the results of further adjustments in model 3 were the same as those in the unadjusted model and were not statistically different (*P* > 0.05).

**Table 5:** Associated analysis of PE and adverse pregnancy outcomes

Pregnancy outcomes	NS + PE(30)	NS (60)	OR(95%CI)	*P*
premature birth				
Model A1:	8.25	1	3.04–22.38	< 0.001
Model A2:	8.25	1	2.73–24.95	< 0.001
Model A3:	7.26	1	2.54–20.70	< 0.001
Foetal birth restriction				
Model B1:	7.43	1	2.64–20.89	< 0.001
Model B2:	5.71	1	1.95–16.71	0.001
Model B3:	4.07	1	1.32–12.51	0.014
foetal distress				
Model C1:	5.67	1	2.07–15.51	0.001
Model C2:	4.26	1	1.49–12.19	0.007
Model C3:	2.69	1	0.87–8.35	0.085
Low birth weight babies				
Model D1:	6.00	1	2.30–15.64	< 0.001
Model D2:	4.77	1	1.77–12.87	0.002
Model D3:	3.42	1	1.21–9.69	0.021
abortion				
Model E1:	2.20	1	0.58–8.29	0.24
Model E2:	4.87	1	1.05–22.71	0.044
Model E3:	4.79	1	0.97–23.65	0.55
maternal outcome				
postpartum haemorrhage				
Model F1:	1.50	1	0.58–3.91	0.41
Model F2:	1.58	1	0.51–4.29	0.43
Model F3:	1.94	1	0.55–6.85	0.30
Haemorrhagic salpingitis				
Model G1:	4.21	1	0.37–48.46	0.25
Model G2:	4.82	1	0.25–93.35	0.30
Model G3:	6.57	1	0.08–549	0.41

Model 1: Unadjusted

Model 2: Adjusted for 24-H urine protein, number of pregnancies, and history of cesarean delivery

Model 3: Further adjusted for total calcium level, uric acid, serum triglycerides, and RBC based on model 2

## Discussion

In the present study, the risk of various adverse foetal outcomes was greatly increased in patients with NS and PE during pregnancy; however, there was no statistical significance in the analysis of maternal outcomes. Although no significant association was found with maternal outcomes in this study, the clinical management of maternal surveillance should be taken seriously. [14, 15]. Maternal outcomes in PE-induced NS have been studied previously; the results were promising, and no deaths were observed [16, 17]. This is consistent with the results obtained in this study and may be related to the fact that the mother was protected from the adverse outcomes by the aggressive medical care she received.

This study showed a significantly higher risk of adverse pregnancy outcomes for the foetus than for the mother. This illustrates that the risk of adverse pregnancy outcomes increased in foetuses after combined PE. This may be related to the causative factors of PE. Placental ischaemia is well known to induce PE and may be a predisposing factor for various obstetric syndromes that coincide with aspects of timing, severity, and disease duration. Persistent placental ischaemia induces various adverse pregnancy outcomes in the foetus [6, 18, 19].

Additionally, in this study we found a correlation between a history of cesarean section and various adverse foetal outcomes. This suggests that a maternal experience of caesarean section increases the risk of adverse foetal outcomes in later life. The adverse maternal-foetal effects of caesarean section in the normal population have been studied previously [20, 21], and adverse maternal and foetal outcomes may be due to the effects of maternal scarring from caesarean section. Although this study was conducted in a population with NS, univariate and adjusted factor analyses showed that a history of caesarean section increases the risk of preterm birth, low birth weight and other adverse foetal outcomes. However, no risk was found for the mother herself, perhaps due to better care.

The prevalent symptom in the NS population is proteinuria [22]. In the present study, the urinary protein level was higher with the combination of preeclampsia than that of common NS, which suggests an interaction between the two disorders. The development of preeclampsia increases urinary protein levels in patients with NS, which increases the risk of adverse pregnancy outcomes in both mother and foetus. In Michal et al., showed that elevated urinary protein levels during pregnancy were predictive of adverse maternal and foetal outcomes [23]. This suggests that elevated urinary protein levels after coexisting preeclampsia may be a contributing factor to an increased risk of adverse foetal outcomes.

This study had several limitations. For example, the patients in this study were selected mostly from pregnant Han Chinese women, which may have produced strong homogeneity and thus prevented the study of the effect of race as a factor on outcomes. In addition, the small sample size was a major limitation. In this study, data from all NS cases from 2012–2021 were selected, leaving only 90 cases after screening. This may have affected the reliability for maternal-foetal outcomes. For example, the combined group in this study did not exhibit significantly different adverse maternal pregnancy outcomes compared to those in the NS group, which may be due to the sample size, and significant results may only appear with a larger sample.Finally, dietary habits, lifestyle, and family history may impact the study outcomes. However, most of these cannot be accurately documented in clinical cases;therefore, more rigorously designed studies are required.

## Conclusion

NS and PE share many similar clinical manifestations, and both often exhibit a mutually reinforcing relationship. The majority of patients with NS have favourable pregnancy outcomes, but the combination with PE at the gestational stage increases the risk of adverse foetal outcomes. Although the harmful maternal effects are unclear, adverse foetal effects may be mediated by the mother. Therefore, it is important to strengthen the monitoring and prevention of PE in women during pregnancy, especially in pregnant women with NS, to focus on the occurrence of PE and provide good clinical care. At the same time, good prenatal monitoring and postnatal care of newborns for pregnant women with NS combined with preeclampsia are necessary to ensure the health of newborns.

## Data Availability

Data and materials were obtained from medical records in hospitals. The datasets used and/or analysed during the current study are available from the corresponding author on reasonable request.

## Abbreviations

NS: Nephrotic Syndrome
PE: Preeclampsia
BMI: Body mass index
OR: Odds ratio
95%CI: 95%Confidence interval
