# Correction: A study of adverse maternal‑foetal outcomes in nephrotic syndrome combined with preeclampsia

**DOI:** 10.1186/s12884-023-06236-7

**Published:** 2024-01-08

**Authors:** Dong Li, Minyi Zhang, Shuxiu Xu, Ziwei Bian, Xiaoli Huang, Guifang Hu, Jing Li

**Affiliations:** 1 Department of Epidemiology, School of Public Health, Southern Medical University, Guangzhou, 510515 Guangdong China; 2 Department of Obstetrics and Gynecology, Nanfang Hospital, Guangzhou, Guangdong China

**Correction: BMC Pregnancy Childbirth 23, 773 (2023)**

10.1186/s12884-023-06073-8

Following publication of the original article [1], the authors reported an error in Funding section. The erroneous fund number (2021A151501184) should be changed to (2021A1515011845).

The original article has been corrected.
